# Gear Hobs—Cutting Tools and Manufacturing Technologies for Spur Gears: The State of the Art

**DOI:** 10.3390/ma17133219

**Published:** 2024-07-01

**Authors:** Norbert Hodgyai, Márton Máté, Gheorghe Oancea, Mircea-Viorel Dragoi

**Affiliations:** 1 Department of Manufacturing Engineering, Transilvania University of Brasov, 500036 Brasov, Romania; norbert.hodgyai@unitbv.ro; 2 Faculty of Technical and Human Sciences Târgu Mureș, Sapientia Hungarian University of Transylvania, 540485 Targu Mures, Romania; mmate@ms.sapientia.ro

**Keywords:** cutting processes, chip formation, tool performance, gear hob, spur gear, gearing processes, gearing technologies, gear hob design, review, gap in literature, future research directions

## Abstract

The present work aims to provide the readers with a bird’s-eye view of the general domain of cylindrical gear manufacturing technologies, including the cutting tools used, and related topics. The main scientific sources are explored to collect data about the subject. A systematization of the scientific works is completed, to emphasize the main issues the researchers have focused on in the past years in the domain. Several specific aspects are investigated: chip-forming process, cutting tool lifetime, the materials used to produce gear hobs, temperature and lubrication, the cutting tool geometry, cutting parameters, design methods, and optimization. Some gaps in the research have been identified, which are mainly related to the gear hob’s design. These gaps, the organization of knowledge, the current requirements of the industry, and the actual socio-economic priorities form the basis for identifying new scientific research directions for the future in the area of spur gears manufacturing technologies and cutting tools. The main output of this work is a frame to guide the development of scientific research in the domain of spur gear production.

## 1. Introduction

The first person who proposed a worm-derived generating tool for spur and worm gear-cutting was the German Christian Schiele. But 1856 was too early to introduce the method in serial manufacturing. After 33 years, the American George B. Grant patented the first known variant of a gear hobbing machine [1]. Finally, in Europe, the first entrepreneur who realized production of gear hobbing machines was the German Robert Herrmann Pfauter from Chemnitz. In this way, gear-cutting started, and the geometric precision of the manufactured gears presented a kind of amelioration. The cutting tool material was still carbon steel because the high-speed steel (HSS) was discovered at the beginning of the XX.-th century. The first official registration of the HSS marked as T1 was completed in 1910 by the company ‘Crucible steel’. Once the HSS was discovered, the cutting tool durability presented a significant increase. However, the problem of the profile conservation was still unsolved. In 1907, the American Hans Baerbalck from Hamilton, Ohio patented an extension for the lathes [2] which allowed the profiling of the relief faces by an Archimedean spiral. Later, with the relieving lathe, the geometry of the gear hob was secured.

Parallel with the evolution of gear hobbing, other gear-generating methods were developed. In 1897, the American engineer and entrepreneur Edwin R. Fellows built the first gear shaper production machine, the famous ‘6-type’, and later, patented a gear shaper cutter grinding machine [3]. It was the first method and machine able to generate internal spur gears, and this method persists almost 99% in machinery nowadays. It is true that a gear hobbing machine for internal gears was created in the early 1970s, but it is applicable only at large circumferences. Finally, in 1913, the Swiss engineer Max Maag patented his gear planing machine and the planing comb. The gear planing machine is the most complex kinematics-presenting machine tool ever created and the most precise; the planing comb is easy to manufacture due to its plain surfaces. But the productivity of this machine is weak, and thus, it remained in the actual machinery in the field of giant gears, where other methods and machines would not be more efficient. Another domain of gear manufacturing was brought to life by the automobile industry, and this is the domain of bevel and hypoid gears with curved teeth. The very special basic types (Gleason, Eloid, and Paloid) were patented, mathematical deductions were not published, and the majority of the deigning formulae were based on practical recommendations. Patent law acted as a shield to protect the mathematical models.

For the spur gears, the development was determined by the evolution of cutting tool materials, and later, the implementation of the numerical control in the structure of the machine tools. Firstly, the stabilization of the manufacturing technology of tungsten carbide inserts and the diversification of these (early 1980s) resulted in the appearance of the cutting insert-endowed gear hobs. The cutting performance increased fascinatingly, but the increased cutting speed and axial feed required more robust machines in order to avoid vibrations. Late, at the end of the 1990s, the physical vapour deposition (PVD) thin layer technologies induced the appearance of a new type of insert with superior mechanical and thermic properties. At the same time, thin layer-endowed carbide gear hobs arrived in the technological infrastructure, which led to a serious increase in the cutting speed and also opened the way to process hardened materials. With this, a grinding quality was nearly achieved. Finally, CNC gear hobbing machines can realize different types of numerically controlled crownings (possible only by grinding). These tooth surface modifications result in a more silent functioning. As can be seen, gear hobbing has come a long way, and has become one of the most popular and efficient gear-cutting methods.

According to the information published on a website [4], “The Global Gear Manufacturing Market is expected to reach USD 39.4 billion by 2025”. The same source mentions as the main methods to produce gears being hobbing, shaping, and grinding, in this order, when it comes to their weight in the global production of gears. The relevance of the general domain of gears (production, manufacturing, control, cutting tools) is revealed also by other specialists [5], who devoted a literature review to the subject. Scholars mention in their publications that a considerable proportion of the gear hobbing is in the domain of gear-cutting, with a huge impact. So, gear hobbing machines represent approximately 50% of gear-cutting machines [4]. Other sources mention that the automotive industry uses the gear hobbing method, in a proportion of 70%, to obtain cylindrical gears [5,6]. The reason for such popularity of gear hobbing consists of its huge productivity in comparison with other cylindrical gear-cutting methods. Basically, there exist four fundamental cylindrical gear-cutting principles: copying using profiled disk mills, shaping using Fellow’s cutters, planing by Maag’s or Sunderland’s planing combs, and finally, the subject of the present paper, hobbing using a gear hob derived from a basic worm.

A simple search on one of the most important scientific databases [7] while focusing on the keyword ”gear” in “the title of articles” provides a list of more than 25,000 references. These statements are enough to emphasize the importance of the subject of the current review, dedicated to the most used cutting tool to manufacture gears—the gear hob—and to the gear hobbing technologies. The present work reveals some of the most important aspects approached by scientific researchers related to gear hobs usage, design, and manufacturing. Identifying some gaps in the achievements obtained so far in scientific research is the basis for formulating some possible future research directions meant to push the domain forward.

The next section of this article focuses on a literature review meant to reveal the main aspects approached by scientists in their published works. The beginning of the published research on gear hobs and gear hobbing is first presented, and then some main aspects of the problems in the gear hobbing processes are categorized, with six subsections, and in the gear hobs, with six subsections. This section highlights the main progress of each topic approached by scientific researchers and published in mainstream journals. Section 3 provides a discussion of the findings presented in the previous section, and is structured in three subsections that aim to systematize the literature, identify some gaps and bottlenecks in the current research, and propose some future research directions. The last section of this article, Conclusion, summarizes the main results of the work.

## 2. A Literature Review

The gear hobs and gear hobbing technologies belong to a very well-established domain. The gear hobbing process was patented in 1835, and the first specialized gear hobbing machine appeared in 1897 [4]. The first article on gear hobbing was published in 1963 [8]. Such a well-established domain, with a long history, is worthy of interest for scientific research. This literature review targets three main aspects related to the gear hobbing process and gear hobs: the cutting process itself, the design of gear hobs, and their manufacturing peculiarities, each of them with several specific issues.

### 2.1. The Gear Hobbing Processes

The gear hobbing process is one of the most complex cutting processes. This is because of the special needs in terms of the kinematics of the machine tools, the complexity of the cutting tool used, the chip-forming conditions, the specific geometry of the gear hob in terms of the cutting angles of the cutter, and so on.

A careful investigation of scientific production may reveal very interesting aspects the scientific researchers have faced.

#### 2.1.1. The Mechanism of the Gear Hobbing Process

The mechanism of the gear hobbing process is considered one of the most complex generating processes. Litvin offers in his book known worldwide ‘Gear Geometry and Applied Theory’ [9] a clear definition and classification of the generating processes. The cylindrical gear surfaces generating process is described here as an example of two-parameter meshing. In this process, the rotation of the gear hob (the first parameter) combined with an imaginary axial shifting determined by the helix parameter forces the cutting edges to reconstruct the basic worm surface which contacts by lines the surfaces of a mobile generating rack. The second parameter is a translation along the axis of the workpiece, the named axial feed, in order to draw the tooth surfaces of the generating rack on their height, which means technologically the machining of the cut gear on whole width. If the cut gear is helical, the helix effect must be compensated for through an additional rotation of the rotary table. The corresponding settings are given in all machine tool handbooks.

**Figure 1:**
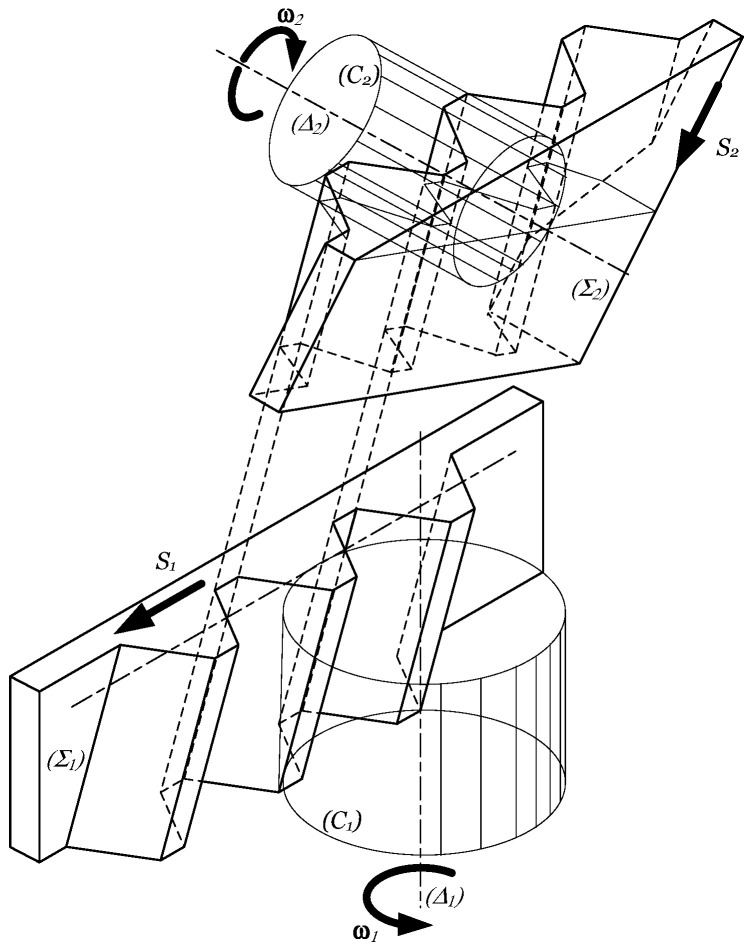
The generation principle of cylindrical gears with gear hobs [10].

The detailed geometric positions are described in a particular way in [10]. The kinematics of the meshing process is shown in Figure 1. This approach reveals the role of the gear hob in the process of involute surface generating with a mobile rack (we consider to mention here that there exists also the generation method with the standing rack, used in the construction principle of Maag’s and Sunderland’s planing machines). The figure describes the case of meshing a helical teethed cylindrical gear. Theoretically, the helical involute tooth surfaces contact along a straight line the generating surfaces of the rack $\Sigma_{1}$. The pitch plane of the rack rolls without slipping on the pitch cylinder $C_{1}$ of the machined gear. Let us consider now another rack, $\Sigma_{2}$, whose teeth are perfectly aligned with the ttooth gaps of rack $\Sigma_{1}$, like pattern and counterpattern.

The basic worm of the gear hob, reconstructed by the cutting edges in the helical motion, is considered an involute worm, which is in fact a helical teethed cylindrical gear, having a huge teeth declination angle, e.g., equal to the complementary angle of the pitch helix inclination angle. As a consequence, it contacts the rack surface by another straight line. Considering both racks, the helicoid involute surface of the basic worm and the helicoid surface of the machined, it can be concluded that the rack tooth surfaces contact by a plane, and the worm surface and machined tooth surface contact the corresponding rack surfaces along straight lines; thus, the worm surface and the machined gear tooth surface contact all the time at the intersection point of the lines mentioned before. In order to mesh the extent of the gear tooth surface, rack $\Sigma_{2}$ is forced to execute a rectilinear motion along the rack tooth direction; the motion denoted is with $S_{2}$. At the same time, rack $\Sigma_{1}$ executes a linear motion of direction $S_{1}$ perpendicular to the axis of the machined gear. Thus, reciprocated meshing of the surfaces is ensured. However, the construction of the gear hobbing machine does not admit the direction modification of motion $S_{2}$. This is set parallel to the axis of the machined gear—in most cases vertically. As a consequence, if machining helical teeth, the vertical feed motion must be summed to the tangential motion of rack $\Sigma_{1}$. This is completed by a differential mechanism in the case of classical machine tools, and by a program when using CNC gear hobbing machines. The main motions and cutting parameters in a gear hobbing operation are as follows:

- Main cutting motion as the rotation about the gear hob’s own axis, characterized by the cutting velocity $v_{c}$, the rotation $n_{2}$, and the angular speed $\omega_{2}$;
- Circular feed motion as the rotation of the blank about its own axis, characterized by the rotation $n_{1}$, and the angular velocity $\omega_{1}$.
- Axial feed motion $s_{ax}$ is the linear motion of the gear hob slider along the workpiece’s axis.

As presented before, the circular feed is dependent on the rotation of the gear hob, the number of teeth of the cut gear, the axial feed, and the teeth inclination angle.

#### 2.1.2. Chip-Forming

The chip-forming at gear hobbing is a complex process, mainly because of the long cutting edge and its differently oriented zones. Due to its significant influence on the cutting forces, and the temperature in the cutting zone, the process of chip-forming raised the interest of specialists both in terms of theory and experiment. Ueda, Y., et al. [11] studied experimentally the chip-forming under the conditions of ultra-high-speed hobbing implemented on a gear grinding machine. To achieve such a high cutting speed, as fast as 2450 m/min, a large diameter gear hob was used with a grinding machine tool.

**Figure 2:**
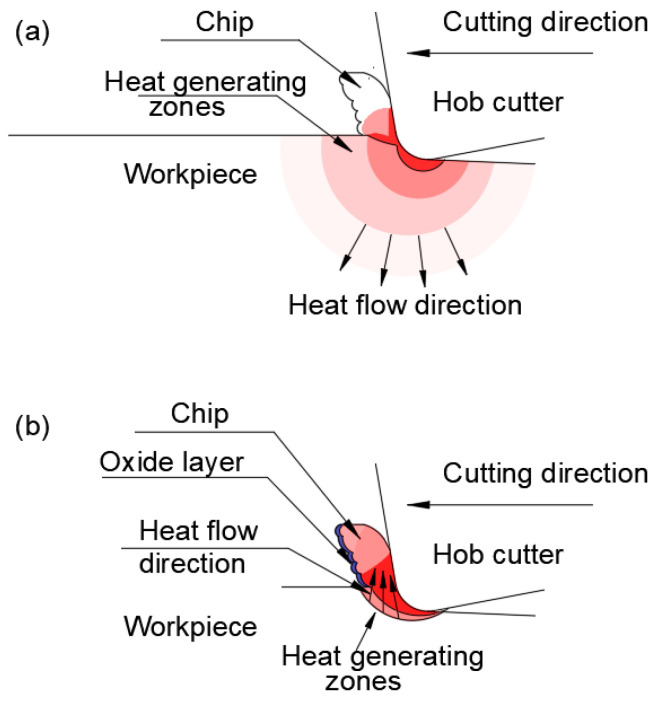
Schematic models for heat flow directions at different cutting speeds: (**a**) low cutting speed, (**b**) high cutting speed [11].

The workpiece material quality was SCM415 (equivalents: GB 15CrMo, JIS SCM415, DIN 15CrMo5) with 610 N/mm^2^ tensile strength and 180 HBW hardness. The gear hobs were built with the WC-Co tungsten carbide. Due to the cutting tool material, experiments were completed in dry cutting conditions. To explore the hardness of the machined surface, tests were conducted using a micro-Vickers hardness tester at a load of 980 mN. After hobbing, the chips and gear surfaces were analyzed using a scanning electron microscope (SEM) and energy-dispersive X-ray spectrometer (EDX). Cross-sectional samples of chips were made using focused ion beam equipment and were inspected with a transmission electron microscope. The residual stress on the gear surface was measured using portable X-ray diffraction equipment. The main findings revealed that the quality and the hardness of the machined surface were high, and the wear of the hob was insignificant. The teeth quality of the surface machined on the gear was illustrated by images taken from the microscope. A comparison of the images taken from samples machined by a cutting speed of 200 m/min, and a feed rate of 0.3 mm/rev and 2450 m/min, 0.3 mm/rev, respectively, emphasizes the difference between the surface layers affected in the two cases. Despite the temperature rising with the cutting speed, the increase in temperature in the workpiece and the hob was small, because most of the heat was removed through the chips. A schematic model of the way the heat migrates and is distributed to the gear hob, workpiece, and chip is also presented for low and high cutting speeds, as shown in Figure 2 [11].

Because of the high temperature of the chips, they are highly oxidized. According to another conclusion, the color of the chip can be considered as a significantly accurate indicator of the temperature during their formation, and thus it can be used as a criterion for the optimization of cutting parameters. The color of the chips can also provide pieces of information to be used for enhancing the geometry of the cutting tool, i.e., the cutting angles. In order to obtain a consistent database for cutting geometry improvement, the chip-forming process was studied with highly sophisticated finite element method(FEM) models [12]. The results were confirmed by the experiments. Furthermore, the action of the chips on the gear flanks was modeled. A virtual machining environment was designed, aiming to study the chip-forming process without the need for experiments [13]. The research aimed to provide a tool to reduce cutting costs by determining the conditions to form un-deformed chips and to predict the cutting forces. The proposed method proved to be much faster and more accurate than the traditional numerical methods. A means to calculate the chip thickness is an analytically determined relationship [14]. Based on some variables (basically, the cutting parameters) and using non-linear regression of the experimental data, a mathematical relationship was developed to determine the maximum chip thickness. It was proved by case studies that the relationship was precise enough.

Despite the numerous research studies developed, the chip-forming process is still to be studied, to better understand its mechanics, and how it can be positively influenced by the gear hob’s geometry. A general approach to chip-forming is presented in [14], regardless of the cutting process, but including gear hobbing. The final goal of the deep research is supposed to be the cutting force decrease.

Regarding the complexity of the chip-forming process during gear hobbing, one can admit that a chip results in a process of oblique cutting with a variable edge inclination angle (this is called the ‘back rake angle’, but in our opinion, the edge position by this angle determines much more the flow direction of the chip than it contributes to the chip-forming conditions; the phenomenon is controlled by the cutting speed and the orthogonal rake angle value). The nose radius in terms of turning can be adopted as the rounding radius by the gear hob tooth, meaning the circular arc edge part that links the side edge with the addendum edge. It is set in usual practice at the standard value, $\rho_{0}=0.38m_{n}$. It also must be mentioned here that the quality of the cut gear dedendum transition profile has an outstanding importance regarding the fatigue resistance and the load capacity of the cut gear. There exists research dealing with the influence of the rounding radius on the properties mentioned before [15]. It must be taken into consideration that the nose radius must be carefully correlated with the cutting parameters, especially the feed, because there exists a minimal limit chip thickness determined by these two parameters under which chip removal is not possible, and the cut surface result will be brittle and scaled. This problem was in extenso studied in [16] for a turning process, but we consider that the results can be admitted in the hobbing process too.

#### 2.1.3. Cutting Forces

**Figure 3:**
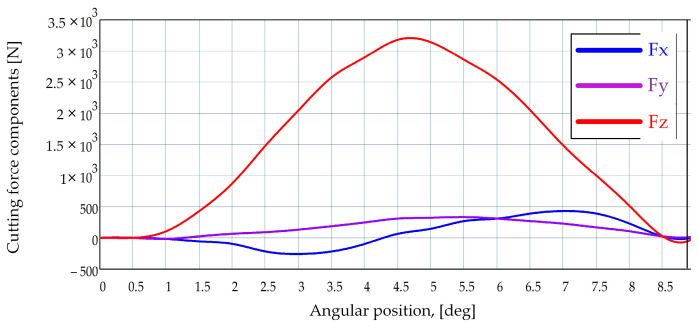
Cutting forces calculation for the hob’s reference tooth during hobbing [18].

Chip-forming is just one of the aspects approached to design the gear hobbing process, another very important one being the cutting forces. An empirical formula to calculate the cutting force is developed, presented, and commented on [17]. It is based on experimental data, and some correction coefficients are used to consider the specific cutting conditions. This is a reason why the precision of the results is rather poor. The cutting factors considered are the maximum thickness of the chip, the modulus, the workpiece teeth number, the bevel angle of teeth, the axial feed, the cutting depth, and the number of teeth of the gear hob. Because of the complexity of the formula, it was further processed by advanced software means, to enhance it [18]. However, the newly obtained formula is applicable only for modules smaller than 30 mm. To find out more about the cutting forces, and to validate the theories related to cutting forces, different systems thought to measure the cutting force at gear hobbing were designed. One is based on the Kistler platform [18]. This allows obtaining by calculi, and based on the measured values, data about each component of the cutting force, as shown in Figure 3. The distribution of the magnitude of each force component is graphically represented depending on the nine successive orientations of the gear hob along its complete rotation. The graph gives a very good image of the way the gear hob is cyclically stressed.

Another set of experiments was designed to perform measurements while hobbing gears made of brass [19]. Experiments were performed to study the similarities and/or differences between dry and wet machining. The monitored parameters were the profile precision, the tooth lead, and the cutting force by the other hand. In order to monitor the stability of the operation, a process capability indicator was introduced, as the quotient between the upper limit and the mean value difference and 3σ. Each experiment consisted of machining a sample of 45 gears. The conclusion was that in terms of gear precision, cutting forces, and process capability, the two are almost the same. It is to be noted that this conclusion cannot apply to other materials, especially if they are of harder machinability.

The cutting forces are used as one of the optimization criteria of the machining parameters [20]. Finding the best values of cutting parameters is often completed experimentally. This is a time-consuming task and generates supplementary costs. To avoid this, a special experimental system was designed, and it was used for the face hobbing of bevel gears.

One can appreciate that keeping under control the cutting forces at gear hobbing is an important objective since cutting forces have an important impact on the wear of cutting tools and they are always a source of vibration. As well, the energy consumption is directly influenced by the cutting forces. A deeper understanding of the way some factors such as cutting parameters, cutting conditions (dry, or wet), and the gear hob’s geometry influence the cutting forces is desirable.

#### 2.1.4. Temperature during the Gear Hobbing Process

It is obvious that the heat produced in the cutting area arises from two sources. The first one is the chip-forming process itself, which is discussed in a previous section. Here the heat is generated by the material deformation and flow. The second heat source is the friction between the different couples of actors involved in the gear hobbing process: the hob’s rake face and the chip, respectively, and the relief face and the machined surface [21]. When it comes to friction, one of the main heat-generating phenomena, it can be reduced using cutting fluids. Their roles are both to cool the cutting area (help evacuate the heat) and to lubricate it, that is, to contribute to the decrease in friction forces. Despite the beneficial effects of the cutting fluids, they must be used under strict control, because of their bad impact on the environment and health. For these reasons, much research has been carried out on lubrication and cutting fluids. The main result was the appearance of bio-lubricants, able to successfully replace the mineral ones [22]. To diminish the bad effect of the cutting fluids, new techniques were developed. The cutting tools equipped with cutting inserts made of special materials (mono or multilayer-coated cemented carbides) can work without cooling. The so-called dry cooling techniques [23] eliminate the usage of cutting fluids or replace them with liquified gases delivered under high pressure (cryogenic cooling) [24]. Another approach aimed at giving up the cutting fluids in studies that targeted the durability of the cutting tools [25]. The study focused on the behavior of gear hobs made of special materials (designed to have high cutting capacity): powder metallurgical high-speed steel PM-HSS S390 and sintered tungsten carbide–cobalt WC-Co K30, under dry machining conditions, when cutting different types of materials. For an easy machinable one, 20 MnCr5, the findings were surprisingly good: the cutting speed could increase up to 350 m/min, and the durability was acceptable, even under dry machining conditions. On the contrary, if the workpiece was of a hard-to-be machined and highly abrasive one—EN-GJS-700-2—the results were disappointing. Even at a cutting speed as low as 50 m/min, the catastrophic wear occurred earlier than expected.

It was proved that the use of lubricants or cutting fluids increases the precision of gear hobbing [26]. A special technique that developed rapidly in recent years is lubrication by Minimum Quantity of Liquid (MQL). This combines the advantages of using lubrication and reducing the bad impact on the environment [25,26,27,28,29,30].

A comparison between dry machining, MQL lubrication, and wet machining (here referred to as flood lubrication) was completed [27]. It was emphasized that the lubricating conditions display particular aspects of gear hobbing, partially different from other cutting processes. Despite that gear hobbing usually needs more abundant lubrication than other processes, the conclusion was that the MQL technique provides the best results: much better than dry machining, and compared to wet machining, the advantages are bigger than the drawbacks.

Another experimental study [28] revealed that the MQL lubrication conditions are very suitable for gear hobbing. A flow rate as low as 100 mL/min offered good cutting conditions: the cutting depth did not have a significant influence on the quality of the machined spur gear. The effect of two non-mineral lubricants was compared in a specially designed study [29]. Eco-friendly lubricants based on synthetic ester and fatty alcohol were used in gear hobbing assisted by MQL. The general conclusion was that the fatty alcohol-based one offered better results, mostly in terms of heat transfer and friction decrease.

However, there is evidence that, in some cases, namely the finishing processes, lubrication cannot at all be eliminated [31,32].

In conclusion, one can say that there is still room for research on the temperature at gear hobbing: the way it influences the precision of the machined part and the wear of the gear hob, resulting in the development of efficient and eco-friendly ways to remove the heat from the cutting zone. As well, further clarification on how the cutting fluid can contribute to chip-forming, chip-breaking, and chip removal is needed. In these terms, inner cooling that feeds the gear hob with cutting fluid from its inside is a possible direction of research.

#### 2.1.5. Wear and Durability of Gear Hobs

The wear and durability of the gear hob are closely connected, the first determining the second. This is why both here, and in the literature, they are treated together. While durability has direct implications for the effectiveness of the gear hobbing process, the wear of the hob directly and strongly influences the dimensional and geometrical precision and quality surface of the machined parts.

In the literature, much research that approaches lubrication [25,26,27,28,29,30] touches on aspects regarding the wear of the gear hob. This is normal, because the lubrication aims, among other things, to keep the cutting edge wear under control.

The gear hob wear is approached from different perspectives in the literature. Research on wear and durability is generally expensive, but when it comes to gear hobs, it becomes even more expensive because of the long time and many resources necessary to spend to obtain a result in experimental research. For this reason, scientific researchers focused on alternative ways to study the subject. An effective method to do that is simulation. Despite the very complex mathematical models needed to describe the phenomena involved, much research has been carried on based on this method.

Based on simulation methods, an interesting theoretical investigation tool was developed [33]. The evolution of the gear hob geometry affected by the wear is analyzed by this theoretical-experimental method. According to the simulation, several specific factors of the gear hobbing process can be predicted with acceptable accuracy, the most important of which are the temperature and cutting forces. In terms of gear hob wear rate, the predicted values are validated by experimental data. A study on the complex process of gear hobbing under conditions of high-speed and dry machining conditions [34] revealed interesting aspects about different forms of wear and how they evolve. The experimental research confirmed the simulated results, so they can be used confidently in the prediction of wear under different cutting parameters. The different ways each gear hob tooth acts to generate the tooth gaps on the machined gear determine different wear modes.

A special and very laborious study [35] was devoted to modeling the different wear types of the successive teeth of the gear hob involved in the cutting process. It showed the high complexity of chip-forming in a gear hobbing process. The form of wear, in terms of cutting theory, was considered the relief face wear, in the transition region between the addendum and the lateral edge, because this is the most stressed part of the tooth. It is defined as flank wear. Experimental research was performed to compare the evolution of the flank wear at a simple HSS-hob and a SUPERTIN-coated one. Results showed that the considered admissible value of the flank wear of 0.3 mm was achieved after a ten-times-larger number of cuts in comparison with the uncoated hob. The evolution of the wear was modeled using a computer program that considers the number of cuts, the equivalent chip thickness, the cutting length, and the cutting speed. Using this prediction model, the goal of the optimization was declared to be the uniform wear of all hob teeth. To achieve this, the vertical (axial) feed was completed with the tangential feed, resulting in a diagonal machining procedure. All experiments were performed with a flying cutter instead of a hob, but the results are accepted.

In gear applications, the designers often use specific tooth profile corrections of the tooth profile, aiming for different purposes. Accordingly, this involves modifications of the gear hob profile. A study [36] explains the way the cutting edge modified shape of the gear hob influences its wear, the shape of the chips, and the distribution of temperature in the gear hob’s teeth. Simulations were used to provide models that can help understand the phenomena and save time devoted to experimental research. The similarity between the simulated results and those obtained experimentally is proved by pictures taken from the simulation and from chips physically obtained through the cutting process.

As mentioned before, research that deals mainly with high-speed gear hobbing [8] makes a very interesting conclusion about the wear of the cutting tool: the wear is not significantly affected by the increase in cutting speed, even if it reaches values in the domain of the ultra-high-speed (up to 2450 m/min).

Research [37] aimed to find out the extent to which the wear of the gear hob is influenced by the lubricant used in machining. The influence of the presence of alumina nanoparticles in the mineral lubricant was the particularity of the study. Two identical gears made of DIN1.7131 material were machined with identical gear hobs, using lubricants with and without alumina nanoparticles. Experimental research proved that the alumina nanoparticles have a beneficial contribution to reducing the craters and the wear of the flanks. The quality of the machined surfaces expressed by the roughness was better, as well.

**Figure 4:**
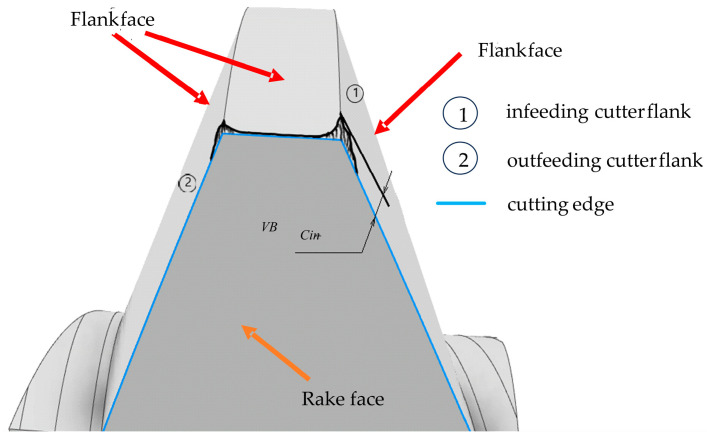
Locations and types of analyzed wears [38].

The influence of the cutting speed on the gear hob wear under different lubrication conditions revealed that the general tendency is that the wear progresses faster when the cutting speed increases. The two lubrication methods were wet lubrication (wet machining—VM) and Minimum Quantity Lubrication (MQL). The cutting speed was varied in four steps within the range of 34.4 to 69.9 m/min. The type of wear analyzed is illustrated in Figure 4 [38].

**Figure 5:**
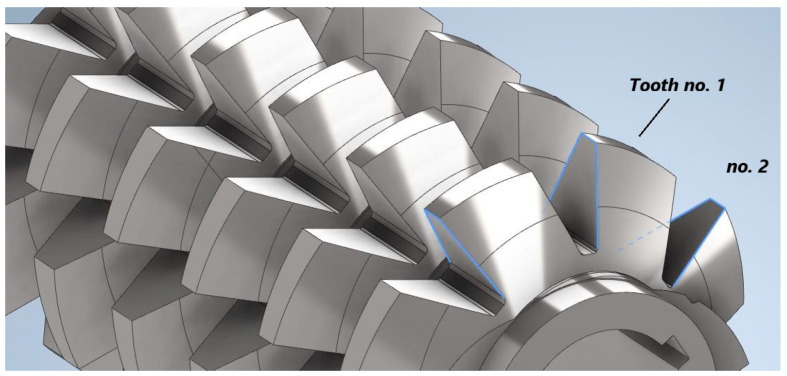
The numbering of the gear hob teeth [38].

The number of teeth affected by the wear was also analyzed, and it was found that the teeth were differently worn depending on their position on the gear hob. The numbering of the teeth is shown in Figure 5. The study revealed that the bigger number of affected teeth reported was always in the case of MQL lubrication. The wear evolution was quite similar for the two lubrication methods, but there was identified a threshold value of the cutting speed of about 50 m/min. where the MQL method does not provide satisfactory results anymore.

Another issue that has a major impact on the wear of a gear hob is the cutting geometry [39]. To study the impact of the cutting angles, a gear hob equipped with cutting inserts was used. Such a constructive solution allows the easy building of gear hobs with different clearance and rake angles. In such a way, each side cutting edge obtains the desired clearance angle. The general conclusion is that the gear hobs equipped with indexable inserts present a much better wear resistance than the conventional ones. Despite the advantages provided by the increased durability of the gear hob, the authors do not mention that such cutting tools can be used only for roughing machining, because of their weakness in terms of profile precision, with bad implications for the geometrical precision of the machined gear, which needs to be finished by other processes.

The local wear of the finishing gear hob raised the attention of researchers [40]. Finishing by hobbing is usually applied as the final processing of the gears roughed by dry machining. The locally different wears of the finishing gear hob cause either low precision of the parts machined or premature tool replacement. Avoiding unbalanced wear can be achieved by an optimal design of the cutting process. To ease that, the local wear process was modeled. It was proved by experiments to be valid, so it became a useful design tool.

Because the tool wear investigation is a very expensive one, mainly in the case of gear hobs, a rapid method to characterize the wear was developed [37]. It was called “flute hobbing” and was first applied and verified for gear hobs made of PM-HSS. The wear of the gear hob was studied for a dry high-speed gear hobbing process.

The wear mechanism, in the case of gear hobbing, depends on the chip-forming model induced by the value of the cutting speed, and the cooling conditions, but firstly it is determined by the resulting tool-in-use geometry. Due to the helix effect, exactly as in the case of turning threads with large pitch values, the side rake and relief angles are strongly different on the attacking and the following flank. Gear hobbing, even in cases of very high cutting speeds, deals with small chip thickness; thus, crater wear on the rake face will never appear. Exactly as in the case of most gear-cutting tools, wear is prominent on the relief faces, due to the small side relief angle values. This is, in the case of gear hobs and generally in the case of gear-cutting tools, unavoidable, because of a compromise to keep the theoretical profile error in the limits of tolerance. However, deformation of the cutting edge occurs, and monitoring is always necessary to follow the evolution of the wear [39,40]. Looking more attentively at Figure 3 and considering that the gear hob’s helix is right-handed, exactly as shown in Figure 4, it is obvious that the left-side edge is the infeeding (attacking), while the right-side edge is the outfeeding (following) edge. Even in the case of Fellow’s cutters, there are significant differences considering the theoretical undetached chip section. Without any doubt, the infeeding edge supports more charge than the outfeeding one; thus, local temperatures are higher on this edge. Due to the large chip section, the cutting forces are also larger, but the tool-in-use relief angle is smaller. Thus, the elasto-plastic deformation of the resulting surface in the radial direction is more significant, and this leads to the accentuated wear phenomena on the relief face. In conclusion, the durability of the infeeding edge is always smaller than that of the outfeeding edge. The wear phenomena are influenced by the position of the analyzed tooth, regarding the rack-gear theoretical line of action. Despite the theoretical approaches, almost all based on [9], that consider a helical tool surface in the meshing process, teeth execute simple rotations about the gear hob’s axis and, thus, have no chance to change their positions regarding the line of action. Special methods and means have been developed and presented to examine the shape of the cutting edge affected by wear [41,42,43]. As a conclusion, teeth situated at the lower and upper limits of the line of action are partially involved in the meshing process, while teeth situated in the middle, in the vicinity of the pitch point, are fully involved in the cutting process.

#### 2.1.6. Other General Aspects of Gear Hobbing Processes

Beyond the particular aspects of the gear hobbing processes, presented above, general issues regarding the gear hobbing itself are widely present in industrial scientific research. Despite the focus of the current work being on the spur gear, it must be mentioned that much research is devoted to other kinds of gears, only a few of which are mentioned here: bevel [44,45,46,47], spiral [48,49,50,51], hypoid [52,53,54,55,56], and spherical [57].

It is more effective to use CNC machine tools’ capabilities to generate sophisticated tool paths necessary to process special gears than using dedicated gear-cutting machines and cutting tools, which need special adjustments. The face hobbing is already a consecrated process for bevel spiral gears. Unfortunately, it cannot be applied for straight-teethed bevel gears, because the teeth profile results were deformed. However, using a six-axis CNC machine tool and a dedicated mathematical model, it is possible, to combine the two motions, one the cutting tool and one of the workpiece, to obtain rectilinear flanks of the teeth placed on a cone-shaped part [44]. Because the five and six-axis CNC machine tools are very expensive, the researchers looked for technical solutions to machine bevel gears with simpler equipment. Combining a three-axis CNC machine tool and a rotary table, it was possible to manufacture a spiral bevel gear [51]. Very sophisticated gears have been machined using a CNC hypoid generator and a dedicated mathematical model. To achieve this, online real-time programming was involved [53]. Using CNC machine tools, even big internal gears can be machined. A special new kind of cutting tool must be used. The problem is to manufacture a cutting tool that is basically a spherical-shaped one, on which the cutting edges must be placed. This is possible using a four-axis machine tool. Furthermore, such a machine tool was transformed, so that by reconfiguring it, along with spherical hobs, elliptical ones and arc surface hobs can be manufactured [57].

As one can see, the CNC machine tools are more versatile and capable of processing different types of gear hobs and complex gears. In terms of manufacturing the gear hobs, CNC machine tools might be useful to machine the relief face of the side cutting edges. Hence, a certain clearance angle can be obtained, which is not allowable by the classical turning process.

In conclusion, in this direction, there is much room for developing research toward new technical solutions for improving the geometry of the gear-cutting hobs.

Coming back to spur gears, some interesting aspects of the gear hobbing process are worthy of being presented. Simulation is an important means to gather pieces of information about phenomena, processes, and other things, using cheap but advanced computerized methods, instead of spending time, money, and resources for this purpose. Thus, a simulation was developed to study the process of gear hobbing spur and helical gears [58]. Based on the computer aided design (CAD) model of the gear, and involving the kinematic of the cutting process, a simulated model was obtained. The result offers 3D geometrical data useful for predicting cutting forces, cutting tool stress, and even previewing the tool wear evolution. Concluding, one may say that the simulation software system can be used for further research.

The simulation was used also to analyze the effect of the relative position (misalignment) of the gear hob and workpiece [59]. The purpose of the study was to identify some means to reduce the noise of the gears used in the automotive domain. A solution was replacing gear shaving with heat treatment followed by gear hobbing, which brings the advantage of the increase in the flank hardness. However, it was found that special attention must be paid to the positioning of the hob worm against the workpiece. Any misalignment has a big negative impact on the geometrical precision of the gear, measured by runout and pitch error. Also, the positioning errors affect the teeth profile. Simulating the gear hobbing process for different inputs regarding the tool positioning, quantitative appreciations could be made on the geometrical errors of the machined gear.

Cutting parameters have an important influence on the general way the cutting process operates. This statement applies even more in the case of gear hobbing, because of the difficult cutting conditions. This is why it is crucial to choose the best values of the cutting parameters. A correct selection of the parameters can be completed based on an optimization process. Much research was dedicated to optimizing the cutting parameters at gear hobbing [60,61,62,63,64]. Among many articles that deal with the subject, an outstanding one provides a method to optimize cutting parameters based on multi-objective optimization [64]. The particularity of the study is that it addressed the small sample problem. The objectives assumed for optimization (evaluation criteria) were the quality of the parts machined, processing time, the total cost of processing, and the carbon footprint (the total carbon emission) considered in all the aspects concerning the processing. The support vector machine (SVM) method was used to generate the first population of parameters, and to obtain the optimal parameters, the ant lion optimizer (ALO) algorithm was applied. In fact, two optimization methods were used and compared. A combination of algorithms and methods—(SVM), and ALO was used in a case study to provide better results in case of the small sample problem than IBPNN/DE, improved the back propagation neural network/differential evolution.

Despite that the spur gears are well established and consecrated, in some situations, they cannot provide sufficient functionality, especially in cases of high-speed gears. Replacing the involute gears (straight teeth) with involute-helix gears brings some considerable advantages: it reduces the noise in operating the gear capable of higher speed and increases the load capacity. This is because the new type of gear put together the advantages of involute and circular gears [65]. Of course, since the manufacturing process is not thought of for CNC machine tools, new dedicated gear hobs had to be designed. A specific mathematical model to describe the concave and convex sides of the teeth made this possible.

Accuracy is a key point in general in engineering and particularly in manufacturing and acquires special importance in the domain of gears. That is why it is widely approached by researchers, whether it is about gears themselves [22,59,66,67,68], gear hobs, or gearing machines [69].

The gearing process can generate errors (profile shape, dimensions, others) in the machined gears [69,70], stress [71], or other defects. The errors might be introduced by the cutting edge of the gear hob, misalignments [59], and the stress can be induced by the heat treatment or by the cutting regime. A very interesting study has investigated the influence of cutting parameters [71]—an investigation on how the hob speed, the axial feed, and the radial cutting depth induce stress at the base of the gear teeth (this is the place where usually cracks appear, or even the fracture occurs). According to the study, this is the sequence of the factors considering their importance in inducing stress in the gear. To measure the stress at the tooth root, the isotropic fixed ψ method was used.

**Figure 6:**
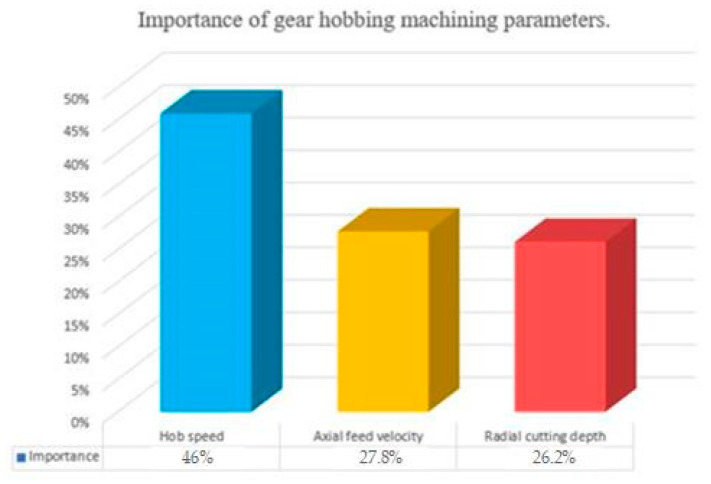
The importance of gear hobbing machining parameters [71].

The proportion of the factors’ influence is shown in Figure 6 [71].

The influence of the rotation accuracy of a gear hobbing machine on the precision of the gears machined is investigated and discussed in [72]. The output of the investigation is very important because it can be used as a tool to select the correct values of the cutting parameters, aiming to obtain reliable gears. The results are all the more useful thanks to the 3D diagrams that illustrate the combined influence of pairs of input factors. Based on these 3D diagrams, optimization according to different goals can be performed.

An important advantage of the CNC gear-cutting machine tools is their capability to change the cutting parameters even during the cutting process if needed. Such a need appears whether severe variations of an output measure occur. To balance such output variations, an input parameter is accordingly adjusted. This is the basic principle of adaptive control [73]. This method was successfully applied to a CNC gearing machine tool [74]. The study was suggested by the relatively low effectiveness of the gearing processes, and the need to improve it. It is known that some input factors may vary during the cutting process (the cutting depth, the workpiece hardness/machinability, and others). To prevent an overload of the machine tools, the cutting parameters are chosen in such a way as to face properly the toughest cutting conditions. This means that for a certain period, the machine tool is underloaded. To bring the output to a normal value, a controllable input is increased. The relationship between the cutting torque (output measure) and the feed rate (controllable input) is exploited. First, a mathematical relationship between the two is established. This is used by a fuzzy controller able to determine (and deliver) the feedback to the machine tool. The feedback consists of an appropriate increase or decrease in the feed rate, according to the amount the torque deviates below or above a preset reference value. Applying this method, a decrease of 30–40% in the machining time is ascertained as being reported to the classic machining, and this can be considered a success.

#### 2.1.7. Gear Hobbing Machine Tools

**Figure 7:**
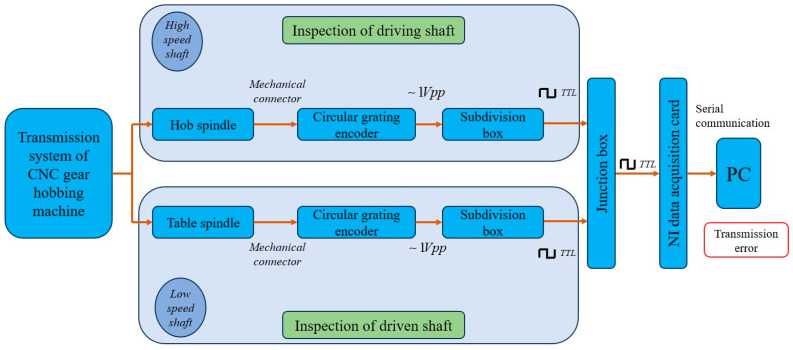
The design principle of transmission error in situ measuring system for gear hobbing machine [77] WPP (Volt Peak to Peak Voltage), TTL (Transistor-Ttransistor Logic, serial communication).

Machine tools are a very important element of the gear hobbing process. They influence decisively the precision, effectiveness, and the cost of the gear hobbing process. Furthermore, their capabilities determine the possibility of generating gears of various shapes. Obviously, the kinematics of the gear-cutting machine tools play a crucial role in the precision of the machined gears. This is a reason the researchers focused their attention on this domain. Studies have been carried out either to evaluate the precision of kinematics [75], and modeling [76], or to trace the transmission errors, aiming to improve them [77]. The novelty of this system consists of removing the uncertainty of the measuring system caused by the place of the encoders, on one hand, and the noise induced by the gears in the kinematic chain on the other hand. Individual circle grating encoders are connected at the ends of the two synchronized kinematic chains: at the spindle of the hob and of the workpiece. The design principle of the measuring system is presented in Figure 7 [77].

The proposed system, dedicated to locating the sources of transmission errors in the kinematic chains, was validated by experimental research. Using it, during the assembling process of a gear hobbing machine tool, adjustments can be applied to remove the transmission error sources. Furthermore, based on the statistical results of the evaluations, measures for improving the precision of the kinematic chains can be drawn.

The CNC machine tools are very agile in generating sophisticated tool paths, so they become important to the specialized machine tools for gear hobbing [68].

To conclude the review of the gear hobbing processes, one may say that any of the topics presented in this subsection leave room (more or less) for new research meant to improve the gear hobbing processes. Based on this assertion, and the gaps identified in the scientific research, some new future research directions will be stated by the end of this article.

### 2.2. Gear Hobs

The gear hobs are the main cutting tools used for manufacturing spur gears. Their most important feature is they ensure the effectiveness of the cutting process. The gear hobs are highly specialized to produce gears, to the same extent as the gear hobbing machine tools. Despite that the gear hobs are very well developed, much research is devoted to their improvement. For a very good understanding of the state of the art in the domain, a systematization of the problems related to gear hobs is necessary. A survey of the literature revealed the most important aspects of the research dedicated to gear hobs, as follows:

- The constructive solution (single block vs. cutting inserts);
- Design;
- Cutting materials;
- The rake face and regrinding;
- Undercuts.

#### 2.2.1. The Constructive Solution

The basic constructive solutions for the gear hobs are single block and composite ones. The single block, or monolithic gear hobs, are made of a single material, and their teeth are cut in a cylindrical raw material. Their main characteristic is the good geometrical precision provided by the generating principle. This is why they are suitable for roughing, but are at the same time the most recommended solution for finishing the spur gears. Since the most used hobs are the single block ones [71,72,73,74,75,76,77,78], they are widely studied by researchers, so they are very present in the literature. The single block gear hobs can be better aligned to the geometrical precision requirements, but display some important disadvantages: relatively low effectiveness, and very importantly, they decrease in geometrical precision with every regrinding. The single block gear hobs are more complex in design and maintenance than the composite ones. Some of their particular aspects will be presented later in this article.

The composite gear hobs (known also as gear hobs with movable cutting inserts) form a narrower domain and they raise specific problems. The composite gear hobs are made of two parts: the main body, and the teeth. The teeth are made of a special material that offers better cutting properties, and they are mechanically assembled on the main body. This constructive solution was adopted because of the advantages they bring, but it is also affected by some drawbacks, as will be shown in the next paragraphs. The cutting inserts, by the material they are made of, are meant to provide better durability to the gear hobs and better stability to their dimensional precision because instead of being reground, they are replaced when worn. They are mechanically assembled on the body, so can easily be replaced when they become worn. The composite gear hobs mainly feature very good effectiveness of machining, and good durability, but have relatively low precision; thus, geometrical errors are transferred to the machined gears.

A general model of a composite gear hob was built [79]. The particular features of the gear hob so defined are as follows:

- A positive rake angle, which provides good cutting conditions through the way the cutting edge approaches the workpiece;
- A planar rake face, tilted against the gear hob’s axis.

Different solutions were proposed to assemble the teeth on the main body: a line of teeth displaced rectilinearly, individual teeth, and even individual inserts for the left and right cutting edges. A computer program was used to determine the correct geometry and position of the cutting inserts. The authors also provided some recommendations/measures to be taken, aiming to increase the precision of the composite gear hobs: the cutting inserts are to be strictly placed in the correct position, modifying the shape of the cutting edge (replacing the straight one with appropriately curved ones), and keeping under control the angles of the teeth.

The composite gear hobs are produced in two constructive solutions: having the inserts placed parallel to the axis of the hob, or aligned to the helical rake face [77]. The second ensures equal rake angles for both the left and right flanks of the teeth, which is an advantage in terms of cutting conditions and inserts’ durability. Whichever solution is chosen, the composite gear hobs suffer from low geometrical precision because of the difficulty of placing accurately the inserts in the desired position and orientation. Furthermore, usually, the cutting inserts have straight cutting edges, and this increases the geometrical imprecision because of the deviation of the straight line from the theoretical curved arc. However, there are available measures to improve the geometrical accuracy [79] by replacing the straight cutting edges with curvilinear ones and adjusting the cutting angles. Adjusting appropriately the orientation of the hob during the machining process, a uniform wear of all the inserts is achieved.

Further studies [80] offer solutions to improve the accuracy of composite gear hobs by modifying the shape of the inserts’ cutting edges, aiming to make the shape approximation more precise and bring it closer to the theoretical shape. An advanced mathematical apparatus is involved, including analytical geometry and mathematical analysis. These special-shaped cutting edges increase the cost to manufacture the cutting inserts and the gear hob itself, but the cutting tool becomes as accurate, so it can be used even for finishing processes.

In conclusion, one may remark that the two types of gear hobs differ not only in the constructive solution but also in terms of their general design, manufacturing, and utilization. Briefly, the single block gear hobs feature good geometrical accuracy, and lower effectiveness (reported to the composite ones), which make them suitable mainly for finishing. Because of their low precision, but high effectiveness, composite gears are usually recommended for the roughing process.

#### 2.2.2. Designing the Gear Hobs

Designing is the starting point in developing a gear hob. It is performed according to the requirements posed by the type of gear to be machined, its main geometrical features, the capabilities of the machine tool they will be used on, and the machine tool used to produce the gear hob, and the list can continue. All these conditions make the designing process of the gear hobs a very complex one. According to its complexity and importance, it has aroused the researchers’ interest to a large extent (note that this section of the article targets exclusively the single block gear hobs).

**Figure 8:**
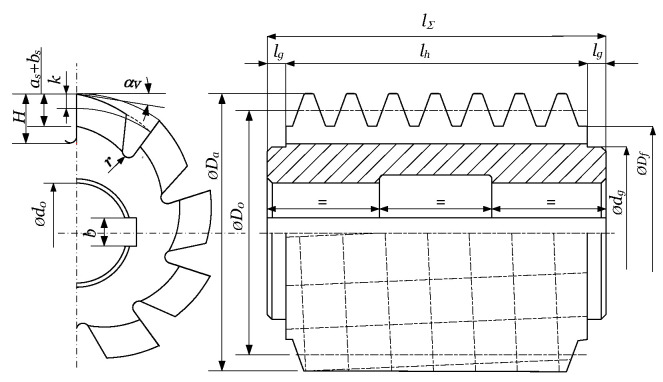
The principal constructive and geometric elements of a gear hob [10].

Any gear-cutting tool must reproduce, through its relative motion referred to the machined gear, the generative part of the technological gear pair. As shown in Section 2.1.1, the generating part of the technological gear drive is a worm, which must be, according to Litvin’s theory [9], an involute worm. The gear hob’s edges must fit the involute worm’s theoretical surface. Teeth must be provided with an adequate cutting geometry. In order to preserve the shape of the cutting edges, a relieving operation is applied. Litvin has also demonstrated that due to the helix effect, involute profile error is unavoidable, and can be kept under control only if the pitch helix angle fulfills $\lambda_{0}\leq 2{}^{\circ}30^{\prime }$. In this case, the difference between an involute worm and a convolute worm is insignificant. The design of a gear hob consists in determining the main geometric elements of the basic worm, followed by the definition of the cutting geometry on the top edge, and finally, the computing of the second order relieve turning cutter and the relieve grinding wheel. The constructive elements of the gear hob are shown in Figure 8 [10].

The design starts from the following initial data [10]: normal module $m_{n}$, number of threads (e.g., teeth) $z_{1}$, normal rack profile angle $\alpha_{n}$ and pitch helix angle $\lambda_{0}$. First, the front section data are computed as follows [10]:

(1) $$\alpha_{t}=\arctan\frac{\tan\alpha_{n}}{\sin\lambda_{0}},\;m_{t}=\frac{m_{n}}{\cos\alpha_{t}}$$

With these, the basic and pitch cylinders diameters result as follows [10]:

(2) $$D_{0}=m_{t}\;z_{1},\;\;D_{b}=D_{0}\cos\alpha_{t}$$

Starting from the normal pitch value $p_{n}=\pi \;m_{n}\;z_{1}$, the axial pitch value results as follows [10]:

(3) $$p_{ax}=\frac{p_{n}}{\cos\lambda_{0}}$$

Now the nearest pitch value must be adopted, which can be set on the existing relieving machine; let us denote this with $p_{ax}^{th}$. The pitch diameter $D_{0}$ must be corrected in such way that the normal pitch must remain unaltered. This results also in the modification of the pitch helix angle. If considering an involute worm, the profile angle of the threading cutters $\alpha_{s}$ must be computed. These are equal to the basic helix angle [7]:

(4) $$\alpha_{s}\equiv \lambda_{b}=\arctan\left(\frac{D_{0}}{D_{b}}\tan\lambda_{0}\right)$$

If deriving the gear hob from a ZN1-type worm, the equivalent normal profile angles $\alpha_{ne}$ are computed through the linearization of the curve obtained by intersecting the thread surfaces with a plane that is perpendicular to the pitch thread [9]. Finally, addendum and dedendum diameters $D_{a}$ and $D_{f}$ result by adding or deducting to the pitch diameter the tool tooth addendum or dedendum heights, in most cases.

The rake face of the gear hob is considered a helical ruled surface, and its leading helix is perpendicular to the pitch helix of the thread. As a conclusion, cutting edges result as the intersection of two helical surfaces. Here, additional corrections are needed.

The relief faces of the tooth are obtained by a helical relieving operation, first turning, and after the heat treatment, finishing by grinding. As a consequence of the relieving, the side relief faces result in conical helical surfaces. After resharpening, the characteristic diameters, including the pitch diameter, will decrease. In this case, the pitch helix angle increases, while the rake face helix angle decreases; thus, they will not anymore be perpendicular to each other. This phenomenon results in the deformation of the cutting edge form, which leads to the deformation of the generating worm while gear-cutting. As a consequence, theoretical profile errors, in the classical concept of the gear hob, are inevitable.

The most challenging issue in designing the gear hobs is the shape of the cutting edge. This is because even if the theoretical shape of the cutting edge can be determined by analytical methods based on the enveloping theory [81], it cannot be physically obtained due to technological restrictions (undercuts, effectiveness restrictions, machine tool kinematics, and others) [82,83,84,85,86,87]. That is why, when talking about the optimal profile shape, the scientists refer to the closest to the theoretic profile, which can be obtained in real manufacturing conditions.

A minimum modification of the tip fillet of the gear hob can avoid the undercuts of the teeth at the machined gear, without significantly affecting its geometrical precision [88]. A parametric model of a gear hob with a modified profile for big modules was created [89]. This allows easily adapting the design to specific technical requirements of the gear to be machined. The second-order cutting tools (relieving cutters for the clearance angle of the gear hob) were also designed. A gear hob was physically realized to prove the validity of the solution. As a side aspect of gear hob designing, the undercuts that occur during the gear hobbing and their effects on the gears machined are also approached in the research [90,91]. This is mentioned here only as a concern of the researchers, not as a directly linked aspect to gear hob design.

#### 2.2.3. Cutting Materials

The gear hobs, mainly the small and medium-sized ones, used to be made of High-Speed-Steel (HSS). With the increasing demand for more and more effective cutting processes, new materials are needed to face successfully the harder and harder cutting conditions: high-speed cutting, the MQL lubricating method, or even dry machining. Also, the need for increased durability is a factor that prompted researchers to seek new performant cutting materials, which did not take long to appear. A study on dry gear hobbing [92] revealed that the powder-metallurgical High-Speed-Steel (PM-HSS) copes successfully with the tough conditions of such machining. A method to determine the correct values of cutting parameters for PM-HSS under dry machining is provided here.

The carbide hobs are very well suited for dry machining due to their high thermal stability, but this material quality seems to be still quite expensive. PM-HSS behaves even much better if coated with thin layers based on (Ti,Al)N, which adds good properties to resist the abrasion and hence to delay the wear [93].

A special technology allows coating the cutting inserts with thin layers of different materials. The technology is called physical vapor deposition (PVD) [94]. What is very important here is that the inserts can be repeatedly ground and again coated, thus significantly extending their operation time. An often-used coating material consists of an oxide based on Mn, Cr, and Fe in roughly even proportions, and important content of Si. A special preparation of the surface to be coated is required, so that the reshaped inserts show good results. Detailed investigation with advanced means of the coated surfaces of the cutting inserts revealed some shortcomings related mainly to the exfoliation phenomenon of the coating layer. A special preparation of the surface to be coated is required, so the coating provides good results. However, the repeated coating of the same cutting insert remains an important advantage.

One can admit that the general tendency in choosing material qualities for gear hobs (and generally for any cutting tool) is oriented to either HSS or carbide, both of them coated with different anti-abrasive thin layers.

#### 2.2.4. The Rake Face and Its Regrinding

**Figure 9:**
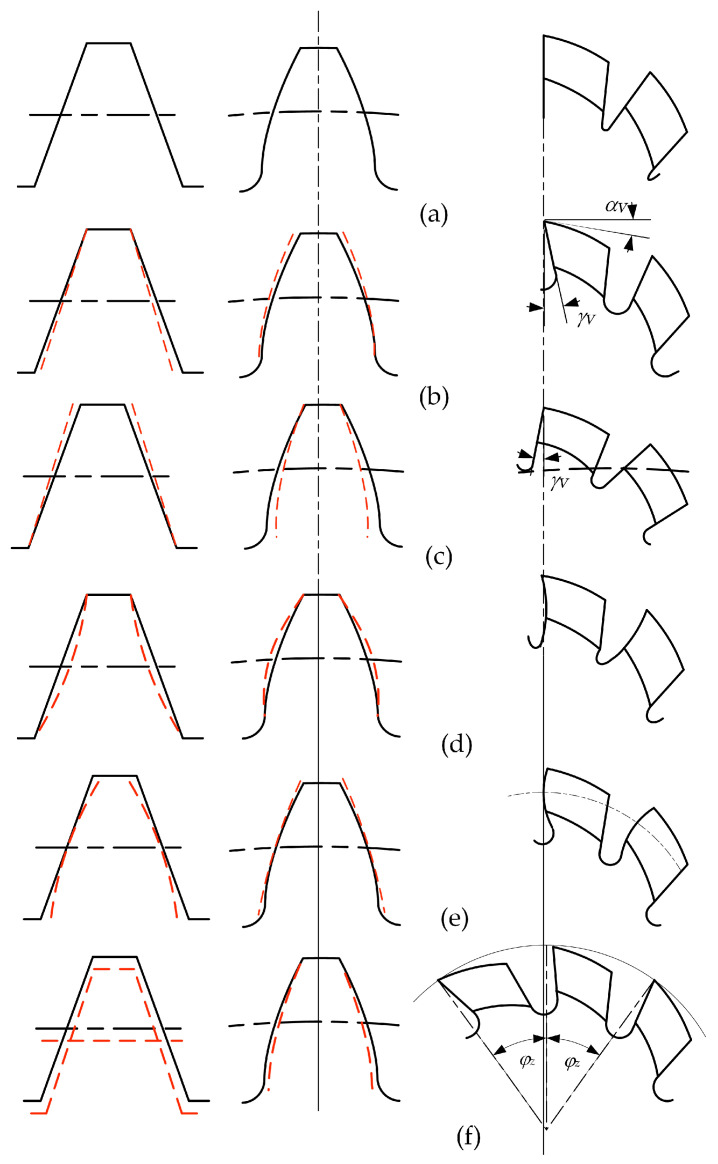
Errors on the tooth of the machined gear due to the lack of accuracy of the gear hob profile. (**a**) 0° rake angle (no errors); (**b**) positive rake angle; (**c**) negative rake angle; (**d**) concave rake face; (**e**) convex rake face; (**f**) unevenness of angles induced by resharpening. The theoretic profile is drawn in a solid line, and the real one in a dashed line [95].

The rake face and rake angle are key issues in the geometry of a gear hob. A very important work that deals with this subject is [95]. Because of the specific construction, the gear hob’s rake face is—in most of the constructive solutions—not a planar one, but helical. This makes determining the shape of the cutting edge a difficult process. According to the cited work, the gear hobs can be designed to have a 0° rake angle, or a positive rake angle. The 0° rake angle is preferred because it is easier to determine the shape of the cutting edge, but this is not the best solution in terms of cutting conditions. Better cutting conditions, when it comes to the cutting process and general behavior of the gear hob, are given by a positive rake angle. This poses a correction of the cutting edge shape to keep the accuracy of the gear hob determining the correct shape of the cutting edge, which in this case is a difficult task. The study [95] also revealed the errors induced by different rake angles in the shape of the machined gear teeth, as shown in Figure 9 [95].

The errors on the tooth of the machined gear are generated by the low precision of the gear hob profile. A 0° rake angle does not produce profile errors at the machined gear. On the contrary, a non-zero rake angle, a concave or convex rake face, and the unevenness of angles induced by regrinding are all factors that alter the correct profile of the gear. The type of error on the gear tooth profile is graphically illustrated for each factor of influence in Figure 9 [95].

A non-zero rake angle causes tilting of the side cutting edges and this almost compromises the possibility of determining the correct shape of the cutting edge, even using advanced means.

Because of specific geometry and technological restrictions, gear hobs are reground exclusively on the rake face. This is why the rake face and regrinding (resharpening, according to some authors) are approached together. Regrinding the gear hob modifies its outer diameter, and thus the helix angle of the tooth, and the helix angle of the rake face, both defined on the pitch cylinder, present contradictory evolutions: while the first increases, the second decreases, and thus, the initial perpendicularity between the tangents and the helices is compromised. The real angle decreases with the sum of the variations of the helix angles mentioned before. The direct effect of this is an alteration of the geometry of the cutting tool with every regrinding. Furthermore, it is impossible to keep the shape of the rake face after regrinding. It was proved that the interference of the grinding wheel and the helical rake face causes the alteration of the shape of the gear hob’s rake face, and hence, also of the cutting edge profile [96,97].

Regrinding is an important concern of the researchers dealing with gear hobs. A study demonstrated that by applying an adjustment to the grinding wheel, the negative effects of regrinding can be minimized [98]. Furthermore, a new circle arc shape is used to replace the theoretical shape of the grinding wheel, so it now can be easily machined on a CNC machine (no need for special interpolations to generate the wheel profile, but only circular interpolation).

Another method to determine the profile of the grinding wheel used to resharpen gear hobs is proposed [99]. A mathematical model was developed to determine the correct profile of the grinding wheel and automatically generate the G code for CNC machining. This method successfully replaces the old-fashioned one, which needs repeated time-wasting adjustments to achieve the desired profile.

The rake face and rake angle with the related subjects—alteration by regrinding, regrinding, the profile of the cutting edges—are generally approached exclusively by geometrical approximations because of the complexity of the surface, and mainly due to the technological restrictions. The conclusion is that there is much room for future research to obtain improved solutions.

#### 2.2.5. Undercuts

The undercut is another issue that generates many problems in gear hobbing. This phenomenon occurs in different situations: when regrinding the rake face of the gear hob, when profiling the clearance face, and when cutting a gear by hobbing. The undercut is never wanted, so special measures have to be taken to avoid it (if this is possible). Once it occurs, the undercut cannot be removed, so the single approach is to prevent its appearance. The undercuts can be prevented mainly by design, but adjustments to the cutting tool can also help the issue. One of the first references to undercuts in the literature dates back to 1983 [90]. Computer-aided modeling and simulation ease the task of revealing the undercut, as a first step to avoiding it. A Visual Basic program is available to create a model of the gear hobbing process. It allows studying how several factors such as the modulus and modification coefficient influence the undercutting phenomena [100].

The minimum number of teeth that can be cut on a gear without undercutting the teeth profile in the specific circumstances of a certain shape of gear hob can be determined by a computer program [101].

An option for new research can aim at new methods of designing and manufacturing the gear hobs able to reduce or even eliminate the undercuts during the machining of the gears. There exist two paths to follow. The first one considers the conical shape of the rake face grinding wheel unchangeable, and thus, the shape of the cutting edges must be computed; then, these edges are rototranslated about the axis of the gear hob, leaning on a conical helix, which results as an effect of the superposition of helical and radial relieving motions. Now, a second relieving grinding wheel profile must be computed. The second solution preserves the ruled shape of the rake face and tries to compute the profile of the rake face grinding wheel. In order to enlarge the setting possibilities, CNC grinding machines may have an emphasized importance here.

#### 2.2.6. Manufacturing of Gear Hobs

The subject of manufacturing can be split into two areas: subjects related to the behavior of the gear hobs during the gear-cutting process, and subjects related to the peculiarities of the gear hob manufacturing technologies. Both domains were already discussed, as reported below:

- Gear hobbing in several aspects, such as chip-forming, temperature in the cutting area and lubrication, cutting forces, cutting parameters, and others;
- Manufacturing (and maintenance—regrinding/sharpening) of the gear hobs.

It can be stated that gear hob manufacturing technology must follow, till a given point, the manufacturing technology of a cylindrical worm. In the case of a monolithic construction (the gear hob is realized from one single blank of raw material), the operations and phases, till the cutting of the flutes, are the same as a worm gear drive’s worm cutting.

**Figure 10:**
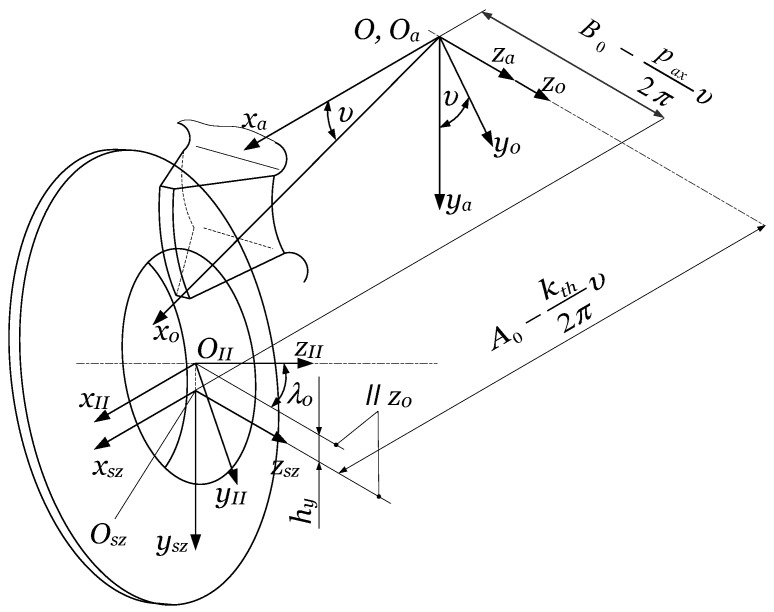
The grinding of relief faces with a disc-type tool [10].

The flutes are realized by milling using a semi-conical disc mill, where the rake face is meshed with the conical part. The profiling of the teeth is realized by relieving. The first phase is the roughing, which is always performed with a profiled turning tool. After the heat treatment, grinding operations occur. The most sensible is the grinding of the side relief faces. There exist two methods: grinding with a disk or grinding with a shaft. If using a disk-type tool, side relief faces cannot be ground on their whole extent, due to the occurrence of interference (Figure 10, [10]).

The side relief faces containing the cutting edges are ground with the conical side of the grinding disk. As one can see in Figure 10, the helical relieving motion is defined by the rotation of the hob υ, the radial feed of the grinding wheel $k_{th}\upsilon /2\pi$, and the axial feed $p_{ax}\upsilon /2\pi$. The axis of the grinding disk is tilted with the angle of the pitch helix $\lambda_{0}$ to the horizontal plane $\left(x_{a}z_{a}\right)$. The reference center of the disk is raised over the horizontal plane by $h_{y}$. It is obvious that, due to the disk radius value and $h_{y}$, there exists mathematically a double infinity of correct solutions for the grinding wheel profile. An optimization for approaching with a circular arc or Bezier curves [100] is the subject of further research.

The resharpening of the gear hob geometrically consists of a series of rotations of the helical rake face about the hob’s axis, with angles corresponding to the relief face wear. Due to this, the tooth basis width decreases while its height remains almost constant, and this leads to the weakening of the tooth rigidity. Practically, it is recommended to stop the resharpening when the rake face fits half of the angular pitch. In conclusion, grinding on the whole tooth surface is not necessary, thus validating the use of the disk-type tool, which assures the necessary cutting speed even at a relatively lower rotation. The disadvantage of the procedure consists of the modification of the profile at every dressing of the disk because its diameter will decrease.

The part of the tooth that cannot be ground must be sunk, in order to avoid interference with the gear blank during the cutting. This can be avoided by applying, from the half of the angular pitch, a second relieving. This is only a roughing operation, characterized by an increased relieving parameter $k_{2}\approx 1.5\;k_{th}$.

**Figure 11:**
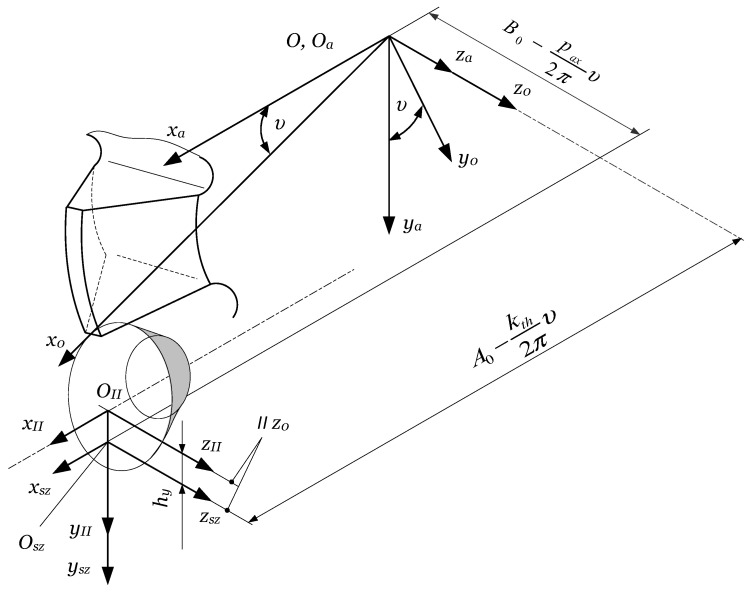
The grinding of relief faces with a disc-type tool [10].

Shaft-type grinding wheels can be also used (Figure 11, [10]). In this case, the whole tooth surface can be ground.

Finally, a very sensitive operation is the grinding of the rake face. An undercut of the rake face by grinding is defined as exceeding the theoretical Radzewich rake face [64]. This is considered a reference due to its simplicity: it is drawn by a straight line, perpendicular to the axis of the worm while describing a helical motion [95]. This is always executed on special gear hob sharpening machines, using the conical face of the grinding disk.

The conclusion that can be stated is that the success of the grinding operation is achieved by using a precise machine tool infrastructure and performant and exact mathematical modeling of the reciprocate meshing surfaces. All these affect the shape of the cutting edge and the shape of the generating worm.

In the case of manufacturing gear hobs with inserts or assembled coil-type insert holders [10], the specific technological problems disappear: here, the geometric precision is assured by the precision and the accuracy of driven axes of the CNC machines involved in the manufacturing process. The precision of the hob is also decisively influenced by the precision of the inserts and the accuracy of their positioning on the main body of the gear hob.

The survey of the literature reveals that manufacturing the gear hobs is a subject less approached by researchers than the gear hobbing process. This statement indicates that there is much room for future research. The concrete-focused research subjects in the domain will be identified and discussed in the next section of the literature review.

## 3. Discussion

### 3.1. A Literature Systematization

Up to this point of the presented research in the current review, the most relevant scientific works were selected. For a clearer bird’s-eye view, they were classified into two large categories: the gear hobbing processes and the gear hobs. In each category, some subdomains were identified, without claiming that the list is exhaustive. For gear hobbing processes, the following were considered most relevant subdomains:

- Chip-forming [11,12,13];
- Cutting forces and torque [14,15,16,17,18,19,20];
- Temperature in the cutting area and lubrication [21,22,23,24,25,26,27,28,29,30,31,32];
- Wear and durability [33,34,35,36,37,38,39,40];
- Other general aspects [41,42,43,44,45,46,47,48,49,50,51,52,53,54,55,56,57,58,59,60,61,62,63,64,65,66,67,68,69,70,71,72,73,74];
- Gear hobbing machines [75,76,77].

As one can see, after the subsection “Other general aspects”, the list continues with one more item which apparently is not directly connected to the previous ones, but which is an important component of the cutting process, so it could not be omitted from the list.

The most populated subdomain is, as expected, the subsection “Other general aspects” because it includes narrow subjects that do not fit either of the previous subdomains.

Some very closely related issues, such as temperature lubrication and wear durability, were joined together in the list.

The large category of *Gear hobs* has been split into five subdomains, as follows:

- The constructive solutions [78,79,80];
- Designing the gear hobs [81,82,83,84,85,86,87,88,89,90,91];
- Cutting materials [92,93,94];
- The rake face and regrinding [95,96,97,98,99];
- Undercuts [100,101,102].

Despite the classification applied, there can be found subjects that easily could be included in either of the two categories. This is because the processes are so closely related to the cutting tools that the domains are difficult to strictly delimit. Such subjects are included in the first category.

Some very particular subjects, such as the hypotheses that are used in gear hobs design, or machining the clearance face of the gear hobs, to mention only two of them, have not been approached in the literature. This fact suggests new future research directions.

### 3.2. Some Gaps and Bottlenecks in the Current Research

Analyzing the literature, the continuous progress of the gear hobs and gear hobbing processes is noticed. Yet, the specialists can observe that some aspects are still not sufficiently studied, and others are not approached at all. Among these can be stated the following issues:

- Still assuming simplifying hypotheses in determining the gear hobs geometry, with bad consequences on the gear hobs’ geometrical precision;
- The CAD/CAM systems facilities are not exploited enough for their entire potential in simulating the cutting processes and designing the gear hobs, observing interferences between the cutting tool and workpiece, and hence undercuts;
- The gear hobs used for finishing are designed and produced exclusively with a 0° rake angle, with bad implications for the cutting conditions—unjustified big cutting forces;
- The gear hobs lose their precision after regrinding because of the decrease in diameter and alteration of the cutting angles and the edge line shape;
- Undercuts do not allow the correct regrinding of the gear hob;
- A gear hob having a planar rake face would be free of some problems related to determining the cutting edge profile and regrinding;
- The problem of low geometrical precision of the composite gear hob persists, and is not studied enough;
- Despite cooling being a sensible issue at gear hobbing, inner cooling is neither applied, nor studied;
- The clearance faces of the gear hob teeth are turned and ground on the relieving lathe; thus, due to the helical relieving process, the side relief angles result in small values;
- Almost not at all exploited the specific features of CNC machine tools in gear hobs manufacturing.

All the issues mentioned above are worthy of being studied, to provide new solutions for improving the gear hobs and gear hobbing processes. Even if some of the proposed re-flection subjects seem to be unsolvable, involving advanced research means, ingenuity and, why, not? the courage to try might offer unexpected favorable solutions.

### 3.3. Future Research Directions

One of the goals of this literature survey was to identify some new future research directions. Some gaps in the scientific research have been mentioned above. According to the classification already presented, some subjects worthy of being further studied are listed below.

#### 3.3.1. General Directions for Research on Gearing Cutting Tools and Processes

The chip-forming process is one of the factors that, along with the cutting tool geometry and the machinability of the materials to be machined, determines the size of the cutting forces. Hence, deep research aiming at a better understanding of the mechanism of forming the chips can help decrease the cutting forces and, indirectly, energy savings.

Despite the negative effect of the thermic deformation of the workpiece, for roughing machining, it would be interesting to know whether either overheating or under-cooling the workpiece leads to lower cutting forces or better cutting conditions.

The temperature and heat removal seem to be kept well under control in green conditions by MQL lubrication and dry machining, even under high-speed conditions. However, high-speed gear hobbing is still worthy of being researched.

CNC machine tools are more and more used in the manufacturing of gears and gear hobs due to their versatility and capability to generate complex tool paths. Their main drawback is low productivity, so research on this subject is needed. A future research direction can target the goal of designing and producing new machine tools that combine the versatility of CNC multi-axis machine tools with the high productivity of specialized gear hobbing ones. The new proposed research directions are in fact extensions of the current research. More challenging research with a much higher level of novelty meant to increase the quality of the gear hobs is needed.

#### 3.3.2. Future Research Directions

A very important issue in designing and producing gear hobs and gears is the geometrical profile errors of the gear hobs, which inevitably are transferred to the gears machined. The main sources of the profile errors are the simplifying hypotheses assumed in determining the profile of the gear hobs and the technological restriction that causes undercuts or other problems. To overcome these problems, some future research directions are stated as follows:

- Adopting new, innovative strategies to determine the gear hobs profile, which do not need simplification, so they lead directly to a real profile that matches perfectly the theoretical one;
- New principles of designing, manufacturing technologies, and regrinding technologies that preserve the correctness of the gear hob precision (shape) after regrinding;
- New geometries of the gear hobs that allow regrinding without undercuts; here can be mentioned the possibility of designing and producing gear hobs with a planar rake face;
- New, or improved technologies to determine the theoretical profile of the gear hob on the rake face positively angled. The result would be a two-in-one gear hob that combines the effectiveness of the roughing gear hobs (positive rake angle) with the precision of the finishing gear hobs (zero-angled rake face);
- New but still machinable shape of the clearance face that ensures an adequate clearance angle of the side-cutting edges.

Once (at least) some of these objectives achieved, important progress in gear hob design and production will be noticed.

#### 3.3.3. Main Requirements for Improving the Gear Hobbing Technologies

Based on the literature review, and the needs of industry and society, three main requirements for improving the gear hobbing technologies have been identified:

- Higher productivity (effectiveness), with the following measures to be taken:
- Applying to an extended scale the high-speed machining;
- Cutting processes with increased feed rate;
- Optimized cutting parameters;
- New cutting materials that offer the cutting tool increased durability;
- Use gear hobs with adequate geometry that allow both roughing and finishing gears by the same gear hob.
- Higher geometrical and dimensional precision of the products: gear hobs, and hence the gears machined, with the following measures to be taken:
- Designing and producing gear hobs with a teeth profile free of errors;
- Designing and producing gear hobs able to preserve the profile precision after regrinding;
- Adequate clearance angle along the entire cutting edge of the gear hob teeth;
- Cutting methods that ensure an even wear of all the teeth of the gear hob.
- Eco-friendly technologies with the following measures to be taken:
- Applying the MQL lubricating, and mostly, dry machining;
- Giving up the mineral lubricants, and replacing them with eco-friendly cutting fluids;
- Applying inner cooling;
- Any measure meant to save energy;
- Approaching the cutting tools (design and production) and cutting processes in an integrated manner that takes into account the carbon footprint along the entire life of the product.

A final remark on future research directions states the need to involve Artificial Intelligence (AI) in the domain of gear hobs and gear hobbing processes in as many research processes as possible, either theoretical or experimental.

## 4. Conclusions

The present work presents an extensive, but not exhaustive, literature review aiming to provide the readers with a bird’s-eye view of the generous domain of gear hobs and gear hobbing technologies for spur and helical gears. The main results of the work are as follows:

1. A systematization of the literature, according to two main areas, gear hobs, and gear hobbing processes, with their subdomains;
2. Identifying some gaps in the literature that need to be filled in by new research;
3. Stating some new future research directions.

The systematization of the literature aimed to group the articles targeting the same narrow subject, so the readers can easily focus on certain problems of gear hobs and gear hobbing technologies. The contents of the review offer information based on the map of the large domain of manufacturing gears. The authors’ view from a distance allowed them to observe some gaps in the literature, some grey and white islands on the map. The first ones need to be further researched so the knowledge can be extended to offer better solutions for certain problems than the existing ones. The white islands either have not at all been explored, or the efforts of the researchers so far have not shown satisfactory results. Identifying the gaps in the past and current scientific research points to the sensible areas inside the technology of spur gears. Based on these gaps, and taking into account the industry’s continuously increasing demands, on the one hand, and the need for sustainable development of the society on the other hand, some possible future directions for research are stated. They target mainly three objectives: increasing the geometrical and dimensional accuracy of the machined gears, higher effectiveness of gear hobbing processes, and an eco-friendly industry of gear hobs and related gears.

In the authors’ opinion, one of the hottest points is the design of the gear hobs. New approaches are needed, so the simplifying hypotheses in determining the cutting edges’ shape are to be removed. Hence, an accurate geometry, identical or at least very close to the theoretical one of the gear hobs, could be achieved, and this would have a direct and positive influence on the precision of the products machined by the newly designed gear hobs. A possible new geometry of the relief face of the gear hobs—considering the advanced and extended facilities offered by the CAD/CAM systems and CNC machine tools—is meant to ensure the proper values of the clearance angle along the entire cutting edge of the tooth, and is susceptible to preserving the shape accuracy of the gear hobs after repeated regrinding, with beneficial effects on the total life of the cutting tools.

There is a belief that these goals can be achieved, taking into account the new capabilities of surface synthesis and the possible involvement in the design process of Artificial Intelligence.

As one can observe, despite the approached domain being a well-established and developed one, there still is much room for new scientific research.

The intention of the authors was to open, based on the current achievements presented in the literature, and on the continuously increasing requirements of the industry, new perspectives for future scientific research on manufacturing spur gears, without neglecting the protection of the environment.

## Data Availability

The original contributions presented in the study are included in the article, further inquiries can be directed to the corresponding authors.
